# Exploring the anticancer potential of green synthesized Zn/Cu nanocomposites from olive leaves against lung cancer

**DOI:** 10.1186/s41065-025-00426-3

**Published:** 2025-04-17

**Authors:** Jing Sun, Shu Mei Tang, Jing Sun, Wei Gao

**Affiliations:** 1 Department Of Respiratory Medicine, University of Health and Rehabilitation Sciences (Qingdao Central Hospital), Qingdao, 266042 China; 2 Department Of PICC, University of Health and Rehabilitation Sciences (Qingdao Central Hospital), Qingdao, 266042 China

**Keywords:** Zinc/copper nanocomposite, Olea europaea, Anti-lung cancer activity, Antioxidant activity

## Abstract

Lung cancer remains one of the leading causes of cancer-related death worldwide, with a significant number of patients succumbing to the disease each year. Olea europaea, commonly known as the olive tree, offers a range of health benefits due to its rich content of antioxidants. In the present study, we have reported the green synthesis of a bimetallic nanocomposite of zinc and copper using the leaf extract of *Olea europae*a (Zn/Cu NCs@ *Olea europaea*). The nanoparticles were characterized using common chemical techniques. The antioxidant activity of Zn/Cu NCs@ *Olea europaea* was evaluated using the DPPH assay. The cytotoxicity and anti-lung cancer activity of Zn/Cu NCs@ *Olea europaea* were investigated using the MTT assay. The results of XRD analysis and FE-SEM imaging showed a crystalline structure for Zn/Cu NCs@ *Olea europaea* with a semi-spherical morphology and an average size of 49.37 nm. Zn/Cu NCs@ *Olea europaea* scavenged the free radical DPPH with an IC_50_ of 363.42 ± 5.02 µg/mL. Furthermore, Zn/Cu NCs@ *Olea europaea* exhibited acceptable anti-lung cancer activity by preventing growth in the cell lines SK-MES-1, A-549, and LK-2 with IC_50_ of 154.00 ± 1.83, 228.83 ± 10.59, and 250.55 ± 8.04 µg/mL respectively. The NPs were inactive against the normal cell lines of HUVEC even at high concentrations. The results of the study indicate that Zn/Cu NCs@ *Olea europaea*, which is green synthesized with a sufficient nano size, can be considered a potent anti-lung cancer agent.

## Introduction

Cancer encompasses a group of diseases characterized by uncontrolled cell growth and proliferation. The incidence of this condition is influenced by a range of risk factors, which can be divided into intrinsic and non-intrinsic categories. Intrinsic factors stem from spontaneous mutations in DNA replication, while non-intrinsic factors include modifiable aspects like smoking, alcohol consumption, nutrient intake, and exposure to carcinogens, as well as endogenous factors such as genetic susceptibility and dysregulated hormone levels. Types of cancer are differentiated based on their tissue or cell origins, showcasing specific anatomical, histopathologic, molecular, genetic, and topographic traits [1, 2, 3]. Lung cancer is prominently diagnosed in both men and women [4]. Lung cancer is categorized into various groups according to histological classification, including mesenchymal tumors, metastatic tumors, epithelial tumors, lymphohistiocytic tumors, and tumors originating from ectopic locations [5]. Lung cancer presents with considerable variability and is predominantly divided into two primary subtypes: small-cell lung cancer (SCLC) and non-small-cell lung cancer (NSCLC). NSCLC accounts for approximately 85% of all lung cancer instances, while SCLC comprises 10–15% of cases. NSCLC is typically categorized into three histological subtypes: adenocarcinoma, squamous cell carcinoma, and large cell lung cancer. Unfortunately, a large percentage of lung cancer cases are diagnosed at advanced stages [6, 7, 8]. Lung cancer carries the highest mortality rate among all cancers in both the United States and China. A robust and successful anti-smoking campaign has played a critical role in reducing the incidence of lung cancer [9, 10].

Individuals often succumb to lung carcinoma as a result of its late diagnosis, typically occurring in its advanced stages. A comprehensive understanding of its pathogenesis, effective early, and the availability of appropriate medications are crucial for the successful treatment of lung cancer. Therefore, timely identification in the early stages plays a pivotal role, especially when screening individuals at higher risk. Additionally, chemotherapy and radiotherapy are essential treatment options [11, 12]. Currently, presents new opportunities for developing controlled drug delivery systems to combat various illnesses, including lung cancer. Nanotechnology can enhance important characteristics such as antimicrobial properties, electrocatalysis, thermal stability, and luminescence, for a wide range of applications in tissue engineering, biotechnology, healthcare, beauty products, food, textiles, and more. The integration of diverse nanostructures into medical practices has been facilitated by the application of nanotechnology in the healthcare sector [13, 14, 15, 16]. The integration of nanotechnology in medicine has advanced the use of various nanostructures in medical settings [17]. Nanoparticles (NPs) are minuscule solid particles at the nanoscale, engineered at the atomic or molecular level to display unique physical properties not found in traditional bulk materials [18]. These tiny particles act as a single unit in terms of their properties. For all materials, there is a critical size or value below which their properties undergo significant changes. Metallic nanoparticles have gained popularity due to their diverse chemical and physical properties, as well as their tunability, which greatly influences their performance, making them ideal materials for various biomedical applications [19]. Bimetallic nanoparticles, composed of two different metals, have garnered significant interest from both scientific and technological perspectives. The selection of metals and their nanoscale dimensions play vital roles in determining the properties of these bimetallic nanoparticles. They are created through various methods that combine different structures of metallic nanoparticles. Bimetallic nanocomposites have recently gained momentum due to their wide range of applications, including anti-corrosion barrier coatings, UV protection gels, lubricants, scratch/abrasion-resistant materials, and more. They also contribute to the production of superior strength fibers and films [20]. The synthesis of bimetallic nanoparticles involves mixing two different metals under controlled conditions, leading to diverse structural and morphological variations. Various combinations of metals, such as noble and transition metals, can produce a wide array of bimetallic nanoparticles. These nanoparticles can be gold-based, silver-based, copper-based, nickel-based, iron-based, platinum-based, or palladium-based bimetallic nanoparticles. Typically, the synthesis process involves either breaking down bulk materials into nanoscale particles or assembling nanoparticles from individual atoms [21].

Natural products are significant reservoirs of biologically active compounds crucial for cancer treatment. Approximately 60% of drugs available today are derived from natural sources [22]. These resources are plentiful and provide promising avenues for discovering therapeutic agents, particularly for challenging conditions like lung cancer, which show limited responses to existing treatment protocols [23]. Medicinal plants have historically been valuable in discovering new remedies for various health issues. They contain a diverse range of secondary metabolites with potent biological activities that can be harnessed for medicinal purposes [24]. The olive tree (Olea europaea L.) is truly iconic in the Mediterranean Basin, offering significant social, economic, and ecological benefits. With six recognized subspecies based on morphology and geography, the olive is versatile, thriving in various climatic conditions, altitudes, and soil types. Its resilience to drought and diverse temperature ranges makes it a valuable crop globally, prominently cultivated in the Mediterranean, Asia-Pacific, North, and South America [25, 26, 27, 28, 29]. Olive leaves have a long history in traditional remedies across European and Mediterranean regions, showcasing a wealth of potential health benefits. Experimental animal studies investigating total olive leaf extracts or their components have revealed a range of therapeutic effects, including hypotensive, anti-arrhythmic, anti-atherosclerotic, hypoglycemic, and vasodilator properties. Additionally, these studies have highlighted the antimicrobial, antiviral, anti-tumor, and anti-inflammatory activities present in olive leaves, underscoring their diverse medicinal potential [23, 30, 31, 32, 33]. In the past, the plant has been used for managing diabetes, malaria, hypertension, respiratory ailments, musculoskeletal conditions, renal issues, urinary tract infections, epistaxis, ocular infections, and alleviating throat discomfort. The historical uses of olive tree-derived products in promoting human well-being span centuries. The notable health benefits attributed to olive tree products primarily stem from the antioxidative attributes present in their constituents. These antioxidant actions might play a role, either directly or indirectly, in various preventative pathways against specific human ailments [34]0.21,22 The phytochemical analysis revealed that *Olea europaea* leaves contain alkaloids, glycosides, phenolics, coumarins, flavonoids, anthocyanins, carbohydrates, proteins, amino acids, tannins, resins, and fats. Numerous investigations have explored the presence of a myriad of phenolic compounds in olive leaves, including hydroxytyrosol, rutin, verbascoside, luteolin-7-glucoside, oleuropein, oleuropein aglycone, ligstroside, as well as other compounds like quinic acid. Generally, among olive cultivars, oleuropein emerges as the most prevalent phenolic compound [35, 36, 37, 38, 39].

In the present study, we focused on the green synthesis of a novel bimetallic nanocomposite of zinc and copper using the leaf extract of *Olea europaea* (Zn/Cu NCs@ *Olea europaea*). The potent antioxidant, anti-inflammatory, and anti-cancer properties of *Olea europaea*, *the* sustainable and eco-friendly approach to the synthesis of green nanomaterials, and the development of a novel drug delivery system for lung cancer treatment were the main reasons for conducting this research. The nanoparticles (NPs) were characterized using various chemical techniques including FE-SEM imaging, elemental analysis of EDX, XRD, FT-IR, and UV-Vis spectroscopy. The antioxidant activity was investigated using a radical scavenging assay. The cytotoxicity and anti-lung cancer activity of Zn/Cu NCs@ *Olea europaea* were studied by MTT assay against the normal cell line of HUV Zn/Cu NCs@ *Olea europaea* EC and human lung cancer cell lines of SK-MES-1, A-549, and LK-2.

## Materials and methods

### Plant material and extraction

The leaves of *Olea europaea* were collected from an olive farm near Qingdao. The plant part was identified by a botanist, and a voucher specimen of WG 1701 was deposited in the School of Life Sciences and Health at the University of Health and Rehabilitation Sciences. To extract from the plant part, 10 g of dried *Olea europaea* leaves were chopped and boiled for 10 min in 100 mL of deionized water. After cooling and filtration, the extract was kept in a cold place before the synthesis of the NPs.

### Green synthesis of Zn/Cu NCs@ Olea europaea

To synthesize Zn/Cu NCs@ *Olea europaea*, 35 mL of the plant extract was added to a 50 mL mixture of Zn(NO_3_)_2_.4H_2_O (0.01 M) and Cu(NO_3_)_2_.3H_2_O (0.01 M) in equal amounts. The pH was adjusted to 8 with NaOH. The reaction mixture was then refluxed for 3 h at 90 °C. Following this, the residue was centrifuged at 6500 RPM for 15 min. The Zn/Cu NCs@ *Olea europaea* were washed three times with deionized water and finally dried at 45 °C.

### Chemical characterization

The FT-IR spectrum of Zn/Cu NCs@ *Olea europaea* was obtained using the PerkinElmer FT-IR spectrophotometer Version 10.6.2 (USA) from 400 cm^− 1^ to 4000 cm^− 1^ using the attenuated total reflectance (ATR) technique; The XRD diagram was obtained in the 2θ scale using an STOE PW2773.00 device with Cu Kα radiation at 45 kV and 40 mA. The diffraction angle (2θ) was scanned from 6° to 80° at a rate of 2°/min. The FE-SEM images and EDX diagram of Zn/Cu NCs@ *Olea europaea* were recorded using a MIRA3TESCAN instrument. The UV-Vis. sectrum was recorded by a UV–Vis spectrophotometer (Jena Speko l2000 spectrophotometer) in the range of 200–800 nm.

### Radical scavenging activity of Zn/Cu NCs@ Olea europaea (RSC) assay

The DPPH assay was conducted to assess the antioxidant activity of Zn/Cu NCs@ *Olea europaea*. To do this, 1 mL of Zn/Cu NPs at varying concentrations (0–1000 µg/mL) was combined with 1 mL of a methanolic solution of DPPH (1 mM). The mixtures were then shaken in the dark for 90 min. Subsequently, the optical density of the mixtures was measured at 517 nm. BHT was utilized as the positive control for this assay. Each concentration was tested in triplicates. The percentage of Zn/Cu NCs RSC was determined using the following equation.

$$RSC\% = \left[ {{{\left( {{A_0} - {A_t}} \right)} \mathord{\left/{\vphantom {{\left( {{A_0} - {A_t}} \right)} {{A_0}}}} \right.\kern-\nulldelimiterspace} {{A_0}}}} \right] \times 100$$

A_0_ is the absorbance at the zero time and A_t_ is the absorbance after 90 min.

### Anti-lung cancer and cytotoxicity of Zn/Cu NCs@ Olea europaea

The cytotoxicity and anti-lung cancer activity of Zn/Cu NCs@ *Olea europaea* were investigated using a standard procedure previously reported [40]. The cytotoxicity of Zn/Cu NCs was tested on normal cells of HUVEC (Normal Primary Human Umbilical Vein Endothelial cells, CVCL_B7UI), and the anti-lung cancer activity was assessed on SK-MES-1 (Lung squamous cell carcinoma, CVCL_0630), A-549 (adenocarcinomic human alveolar basal epithelial cells, CVCL_0023), and LK-2 (Human lung squamous cell carcinoma, CVCL_1377) cell lines obtained from Shenzhn Bike Biotechnology Company, China. To begin, each cell line was cultured in appropriate media, humidity, temperature, and atmosphere. Subsequently, the cells were transferred to a 96-well plate containing medium and exposed to Zn/Cu NCs@ *Olea europaea* at varying concentrations (0–1000 µg/mL) for 24 h. The viability of the cell lines was then determined using an MTT assay, with each concentration tested in triplicate. Doxorubicin 2 µM) was used as the positive control.

### Statistical analysis

The results were analyzed using Origin software with the one-way ANOVA method. The data has been reported as mean ± SD.

## Results and discussion

### Chemical characterization

**Fig. 1:**
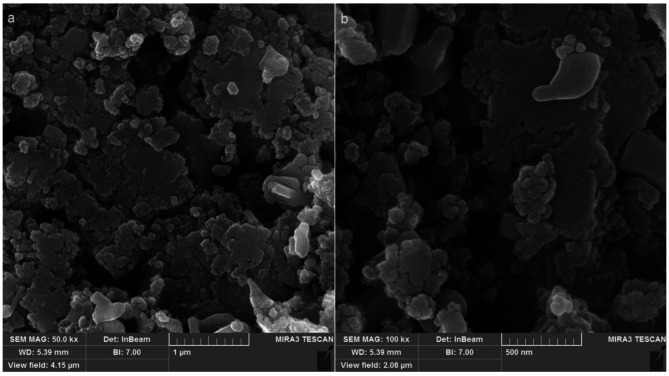
The FE-SEM images of Zn/Cu NCs@ *Olea europaea*

The morphology of Zn/Cu NCs@ *Olea europaea* was analyzed through FE-SEM imaging. The images can be seen in Fig. 1. The results indicate that the nanocomposite takes on a semi-spherical shape with an average size of 49.37 nm, smaller than the size of a chemically synthesized Zn/Cu nanocomposite [41]. Like other metallic nanoparticles, Zn/Cu NCs@ *Olea europaea* exhibit a tendency to aggregate. This property is commonly observed in this class of materials, as shown in previous studies on the green synthesis of metallic NPs [42, 43, 44]. It appears that the presence of organic compounds from the plant extract is responsible for this characteristic of NPs.

**Fig. 2:**
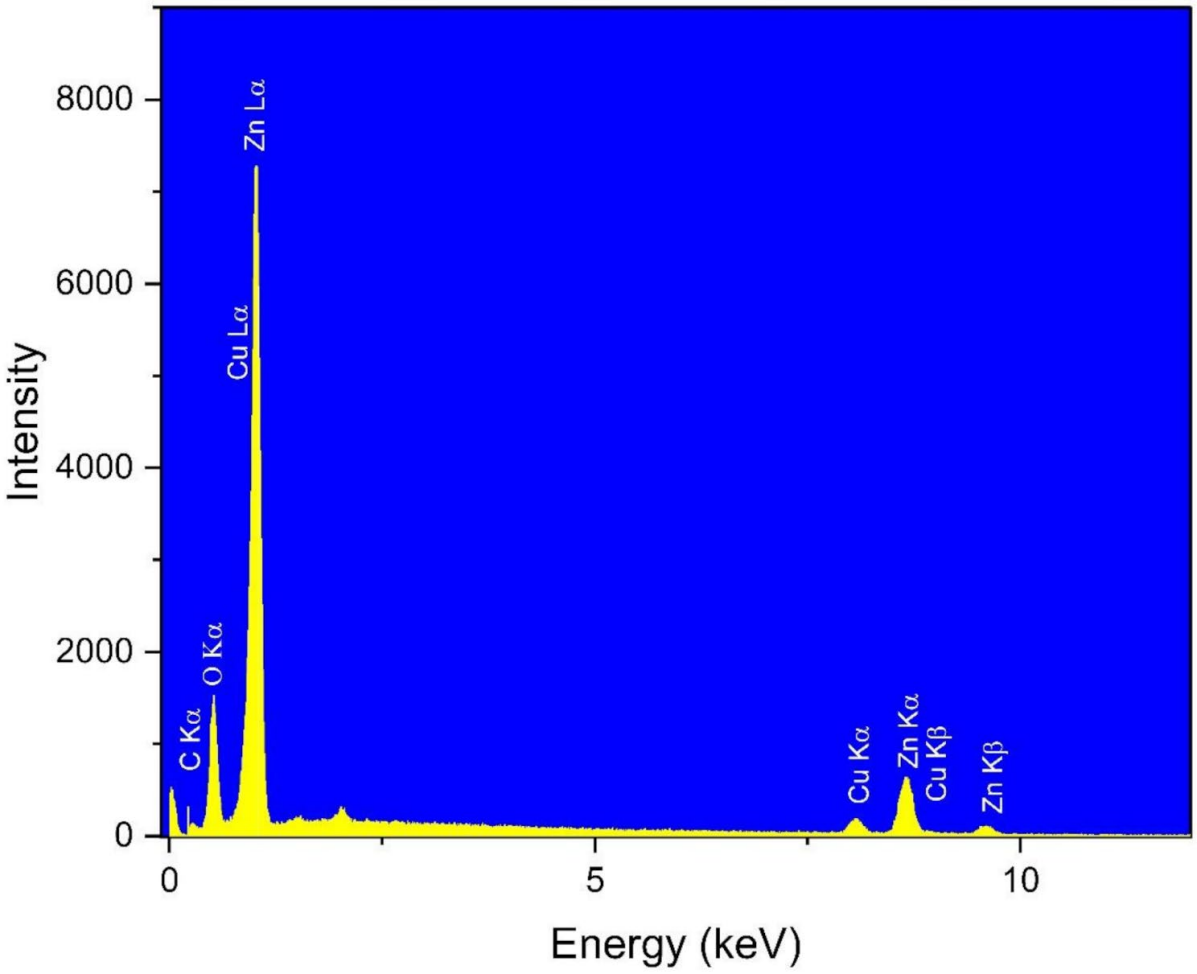
The EDX diagram of Zn/Cu NCs@ *Olea europaea*

The elemental analysis of Zn/Cu NCs@ *Olea europaea* was studied using energy dispersive X-ray analysis (EDX), which is an efficient qualitative method to investigate the elemental structure of nanomaterials. The results are graphed in Fig. 2. The presence of copper and zinc in Zn/Cu NCs@ *Olea europaea* is confirmed by the signals at 0.92, 8.03, and 8.87 keV for Cu Lα, Cu Kα, and Cu Kβ respectively; and the signals at 1.02, 8.65, and 9.60 keV that belong to Zn Lα, Zn Kα, and Zn Kβ. Additionally, signals of carbon and oxygen appeared at 0.24 and 0.54 keV. Hitkari et al. have reported similar signals for a bimetallic nanocomposite consisting of zinc and copper [41].

**Fig. 3:**
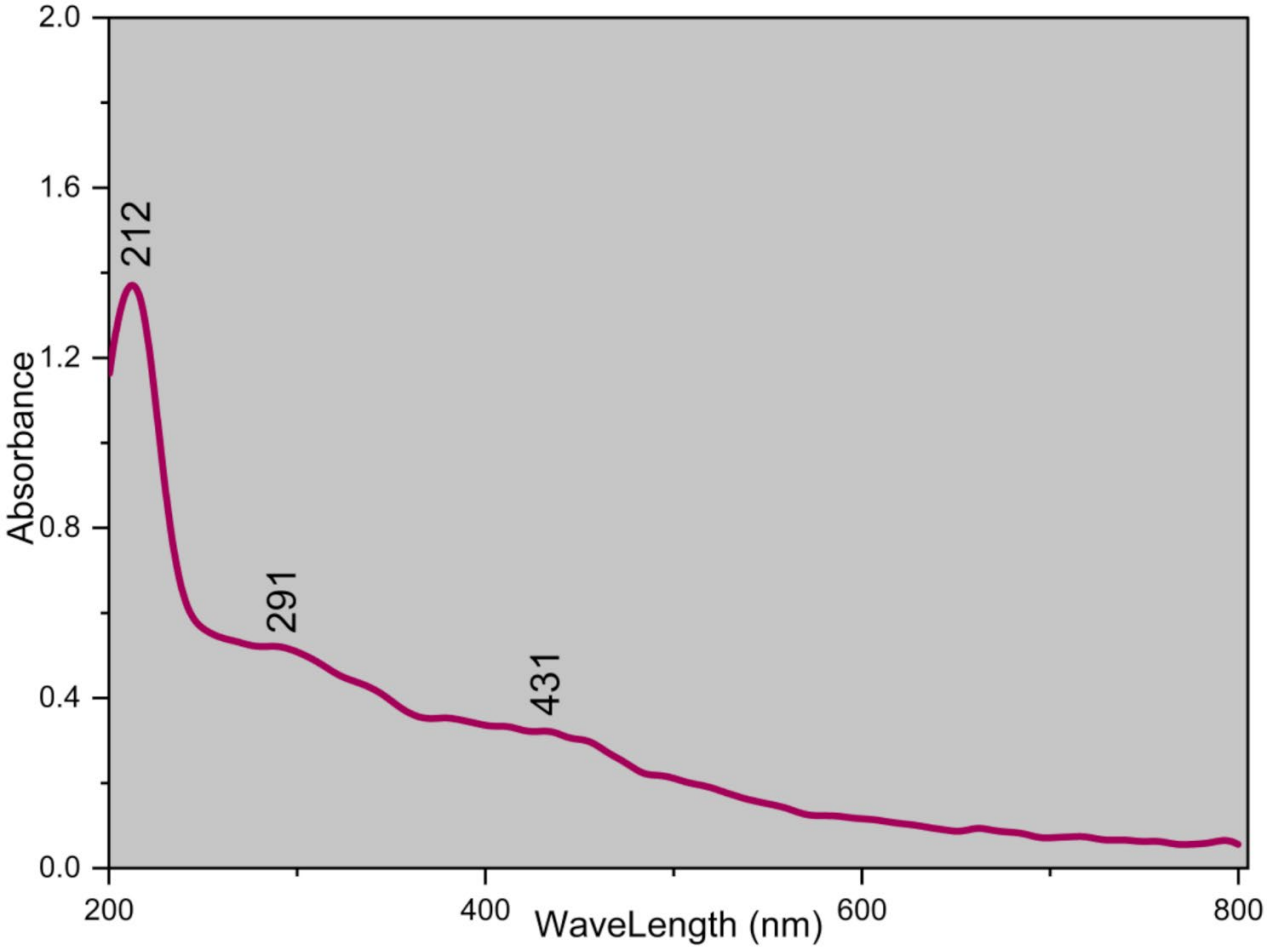
The UV-Vis. spectrum of Zn/Cu NCs@ *Olea europaea*

Figure 3 displays the UV-Vis. spectrum for the surface plasmon resonance (SPR) of Zn/Cu NCs@ *Olea europaea*. Surface plasmon resonance (SPR) on green-synthesized metallic nanoparticles is a fascinating phenomenon that arises from the collective oscillation of free electrons on the nanoparticle surface when excited by light. The use of green synthesis methods, such as those involving Olea europaea leaf extract, presents a sustainable approach to producing metallic nanoparticles with unique SPR properties. According to the results, the bands at 212, 291, and 431 indicate the formation of Zn/Cu NCs@ *Olea europaea*.

**Fig. 4:**
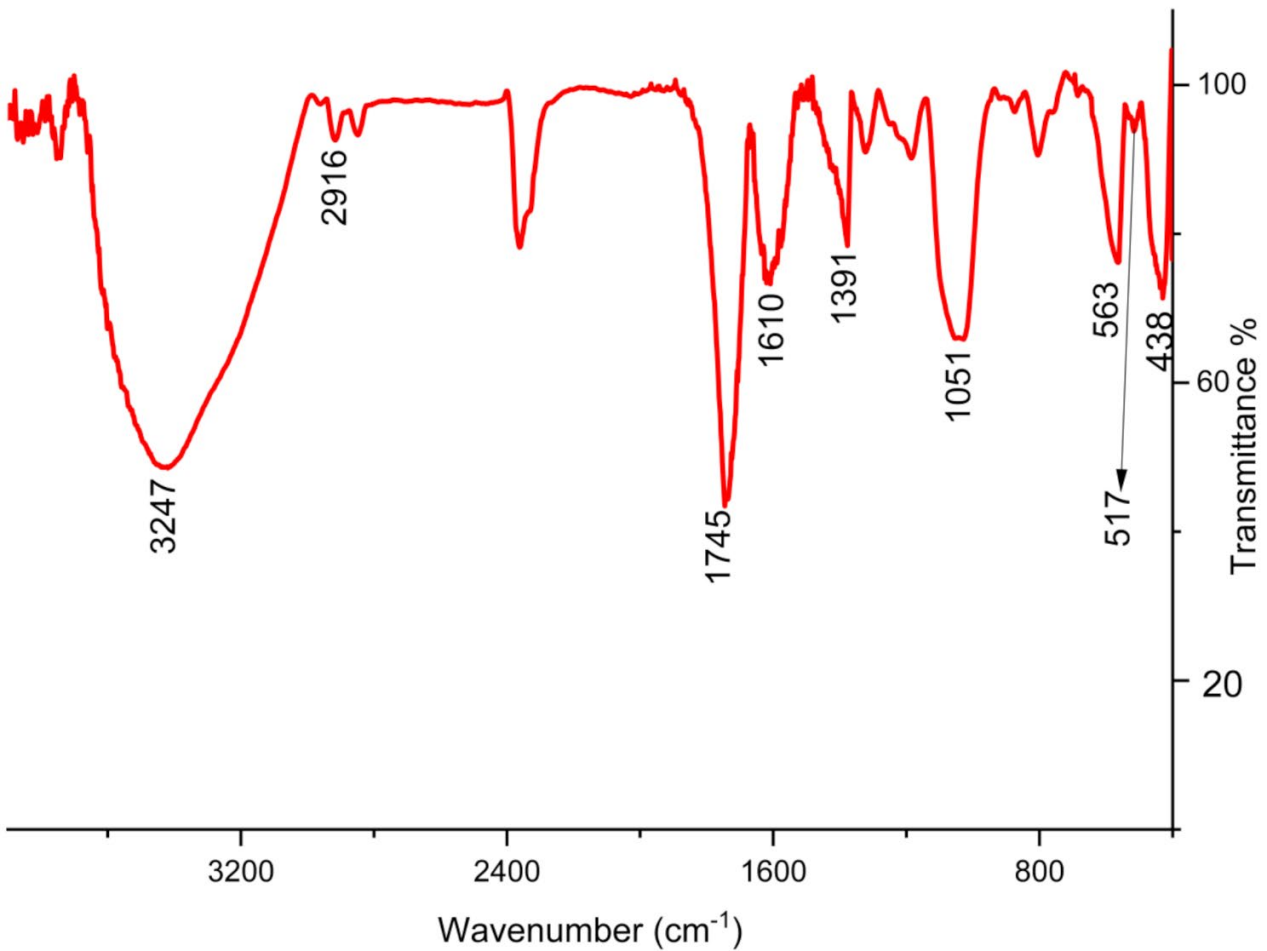
The FT-IR spectrum of Zn/Cu NCs@ *Olea europaea*

FT-IR spectroscopy is a reliable method for assessing the presence of various bonds in metallic NPs. This technique allows for the screening of the metallic bonds and organic functional groups. The FT-IR spectrum of Zn/Cu NPs is shown in Fig. 4. The peaks at 438, 517, and 563 cm^− 1^ are attributed to metal-oxygen bonds, which have been previously reported for copper and zinc nanoparticles. Other bands at different wavenumbers such as 1051, 1395–1745, 2916, and 3247 cm^− 1^ correspond to organic functional groups like C-O, C = C, C = O, C-H, and O-H from the secondary metabolite of *Olea europaea* extract. These groups are known to act as reducing and capping agents in the green synthesis of Zn/Cu NCs@ *Olea europaea*, and they are attached to the surface of Zn/Cu NCs@ *Olea europaea.*

**Fig. 5:**
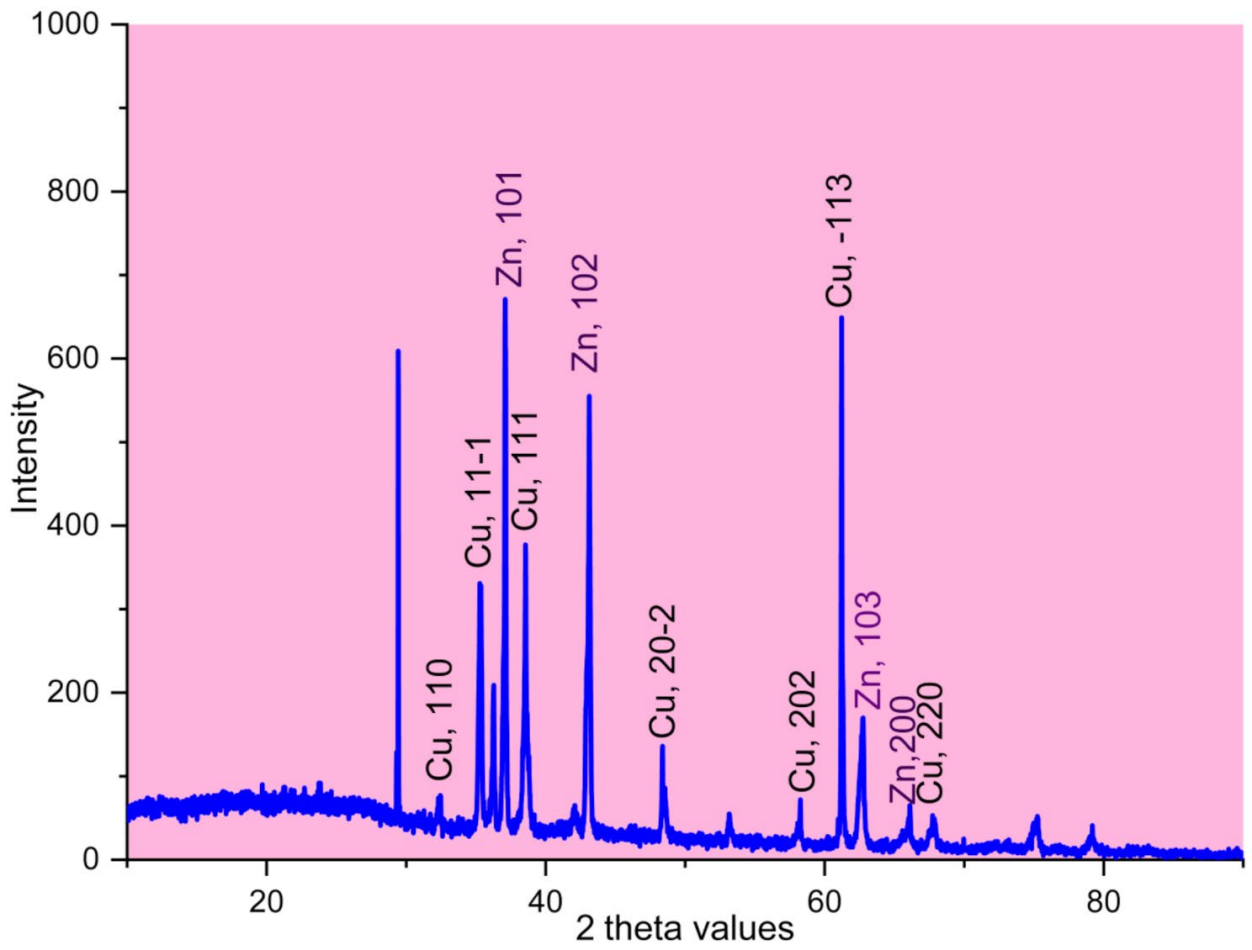
The XRD diagram of Zn/Cu NCs@ *Olea europaea*

XRD analysis is a common method used to study the crystallinity of materials. The XRD graph of Zn/Cu NCs@ *Olea europaea* is shown in Fig. 5. The presence of different signals at 2 theta values indicates a crystal structure for Zn/CuNPs. The signals correspond to CuO and ZnO with a slight shift. Specifically, the signals at 32.47 (110), 35.35 (11 − 1), 38.55 (111), 48.37 (20 − 2), 58.30 (202), 61.21 (-113), and 67.62 (220) match to PDF card No. 96-901-6327 for CuO. The other signals of 36.16 (101), 43.12 (102), 62.74 (103), and 66.10 (200) are well matched to JCPDS card No 136-14511 for ZnO. These results are consistent with a previous study on the synthesis of zinc/copper nanocomposites [41].

### Bioactivity evaluation of Zn/Cu NCs@ Olea europaea

#### Antioxidant activity of Zn/Cu NCs@ *Olea europaea*

**Fig. 6:**
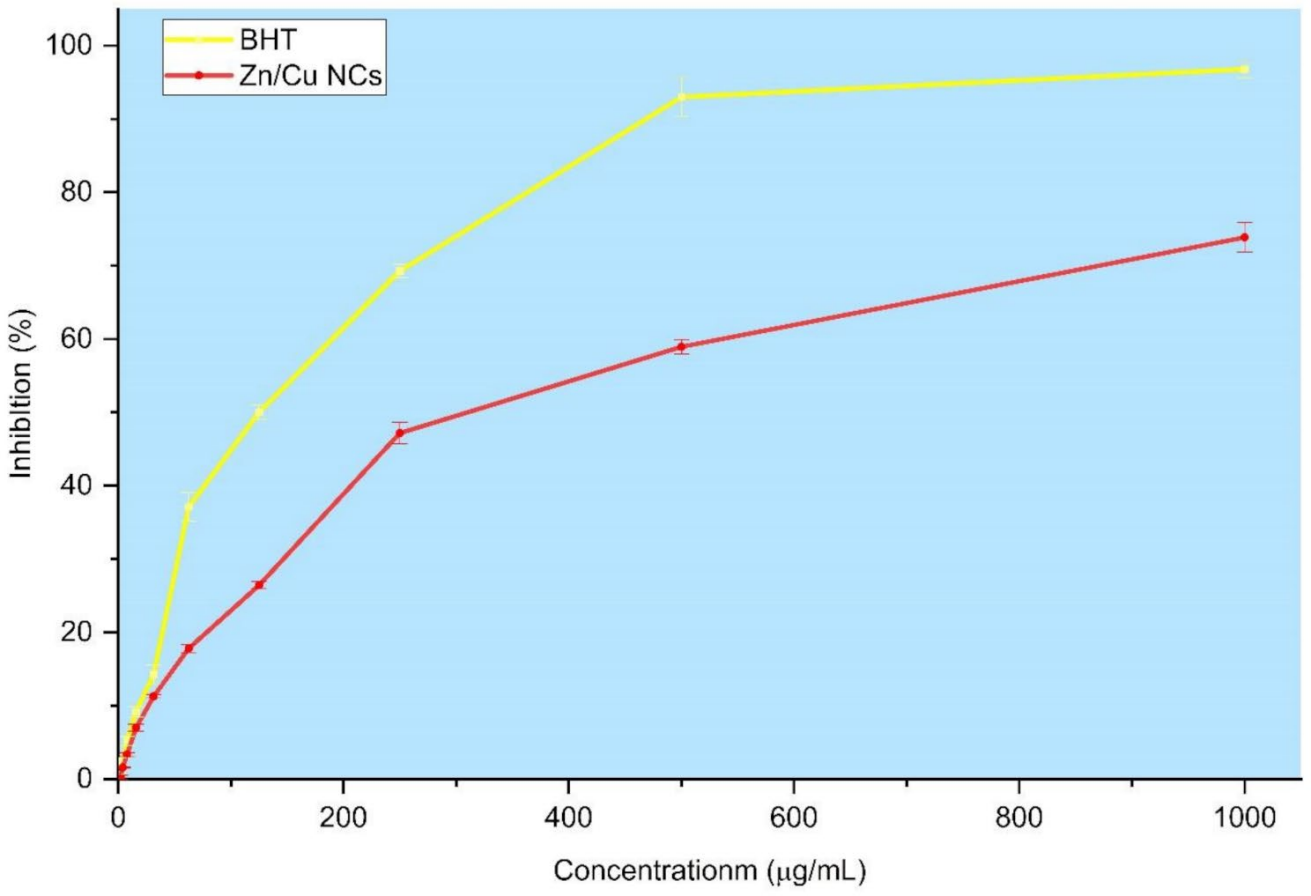
The antioxidant activity curve of Zn/Cu NCs@ *Olea europaea* and BHT at concentrations ranging from 0–1000 µg/mL for scavenging free radicals of DPPH

The DPPH assay is a common method used to evaluate the antioxidant activity of nanoparticles. In this method, the material scavenges the free radical DPPH, causing the purple color to change to yellow. The more antioxidant activity present, the greater the color change. The antioxidant activity of Zn/Cu NCs@ *Olea europaea* is depicted in Fig. 6, with a comparison made to the antioxidant activity of BHT as a positive control. The results indicated a dose-dependent activity for both samples. An IC_50_ value of 363.42 ± 5.02 was obtained for the scavenging of DPPH by Zn/Cu NCs@ *Olea europaea*, while a value of 132.95 ± 6.10 was calculated for the IC_50_ of BHT. Anticancer activity and antioxidant activity are closely linked in several ways. Antioxidants play a crucial role in combating oxidative stress by neutralizing free radicals that can damage cells and potentially lead to cancer formation. Many natural antioxidants found in fruits, vegetables, and other foods have been shown to possess anti-cancer properties by reducing oxidative damage to DNA and other cellular components. Additionally, some compounds with anticancer properties also exhibit antioxidant effects by scavenging free radicals and protecting cells from oxidative stress. Therefore, the relationship between anticancer and antioxidant activities underscores the importance of maintaining a balance in cellular redox status to promote overall health and potentially reduce the risk of cancer development. For metallic nanoparticles, the ability to trap reactive species such as radicals, ROS, and RNS is the primary reason for their antioxidant activity [45].

#### Anticancer and cytotoxicity activity of Zn/Cu NCs@ *Olea europaea*

The development of effective cancer treatments poses challenges, particularly due to limitations in selecting suitable therapies and concerns about the toxicity of conventional chemotherapy drugs. Hydrophobic chemotherapy drugs have limitations in their solubility in water, making it challenging to administer them in high doses without causing excessive toxicity to normal cells [5]. Nanotechnology offers a potential solution to certain challenges in cancer treatment. The unique features of nanoparticles, such as their small size, magnetic and optoelectronic properties, and specific atomic arrangement, make them a deal for targeted drug delivery. Nanobiotechnology has made significant progress in cancer treatment by harnessing these nanoparticle characteristics [46, 47]. When nanoparticles are administered intravenously, they come into contact with plasma proteins and lung lining fluid proteins, leading to the formation of a ‘protein corona’ around their surfaces. This protein corona plays a role in the rapid clearance of nanoparticles by the mononuclear phagocyte system (MPS). Two critical properties of nanoparticles that are significant in cancer treatment are their uptake by cells and their ability to release bioactive ions [11]. Nanoparticles are typically absorbed by cells through mechanisms such as endocytosis and are commonly transported to endolysosomes. Once there, they can either enter the cytosol or be expelled from the cell. Oxidation of nanoparticles can lead to the liberation of metal cations, which could have unexpected consequences, particularly in the instance of bimetallic nanoparticles. The use of nanoparticles in cancer therapy shows great promise in addressing certain drawbacks of conventional chemotherapy and improving treatment efficacy while reducing harm to healthy tissues [48, 49, 50].

**Fig. 7:**
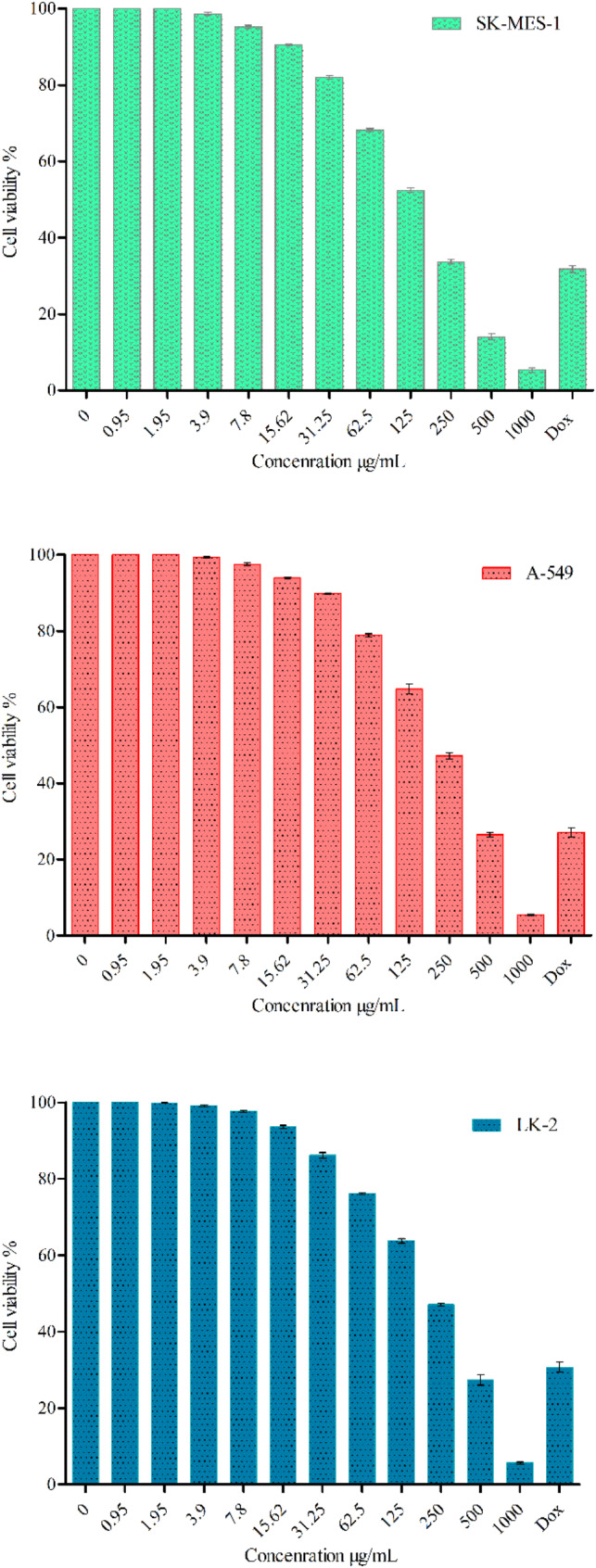
The anti-lung cancer activity graph of Zn/Cu NCs@ *Olea europaea* at concentrations ranging from 0–1000 µg/mL, compared to the positive control of doxorubicin (Dox) at 2 µM. The graph demonstrates the activity against lung cancer cell lines LK-2, A-549, and SK-MES-1

**Fig. 8:**
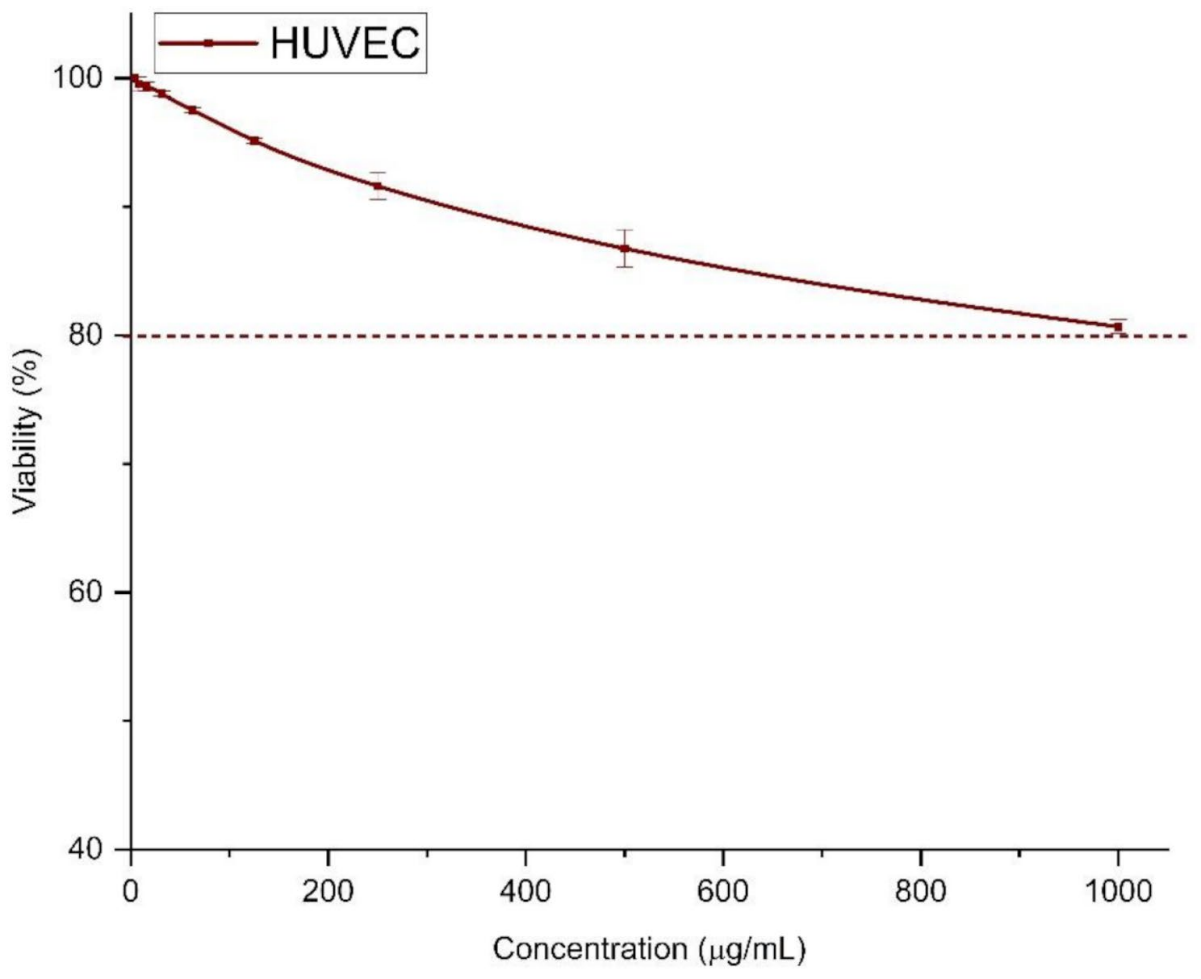
The cytotoxic effects of Zn/Cu NCs@ *Olea europaea* at concentrations ranging from 0–1000 µg/mL on the normal cell line HUVEC

The anti-lung cancer activity of Zn/Cu NCs was investigated against the human cell lines SK-MES-1, A-549, and LK-2. The results are displayed in Fig. 7. For all selected cell lines, Zn/Cu NCs@ *Olea europaea* exhibited dose-dependent activity. The highest inhibition was observed against SK-MES-1 with IC_50_ of 154.00 ± 1.83 µg/mL followed by A-549 (IC_50_ = 228.83 ± 10.59 µg/mL) and LK-2 (IC_50_ = 250.55 ± 8.04 µg/mL). The results indicated that the anti-lung cancer activity of Zn/Cu NCs@ *Olea europaea* was more pronounced at higher concentrations of 250 µg/mL compared to the positive control of doxorubicin (2 µM). The cytotoxicity of Zn/Cu NCs@ *Olea europaea* was evaluated against the normal cell line HUVEC (refer to Fig. 8). The findings revealed a non-toxic effect even at the highest concentration of 1000 µg/mL, with around 80% cell viability in HUVEC cell lines. Therefore, the results of this study demonstrate that Zn/Cu NCs@ *Olea europaea* have a strong anti-proliferative effect on human lung cancer cell lines. Several recent studies have examined the anticancer activity of Zn/Cu bimetallic NPs. For instance, Zhou et al. investigated the anti-breast cancer properties of chemically synthesized Zn/Cu bimetallic nanoparticles against a 4T1 breast cancer cell line [51]. Additionally, Zn/Cu NPs synthesized using Lonicera *caprifolium* plant extract demonstrated activity against the MCF-7 breast cancer cell line of MCF-7 cell line [52]. Recent studies have highlighted the potential of zinc-based nanocomposites, specifically zinc metal-organic frameworks (Zn-MOFs), in cancer treatment. These materials are being investigated for their ability to combine cancer diagnosis and therapy [53]. For example, Zn-NMOFs coated with folic acid functionalized chitosan have shown promise in delivering chemotherapeutic agents like doxorubicin to breast cancer cells. They have demonstrated low toxicity and high efficacy in inducing apoptosis and autophagy [54]. Copper-based nanomaterials have also attracted attention for their antitumor properties. They are known for their ability to induce oxidative stress and initiate programmed cell death in cancer cells while sparing healthy cells [55]. Both Zn and Cu nanocomposites offer unique advantages in cancer therapy, representing promising avenues in oncology. Each contributes distinct benefits to cancer diagnosis and treatment. Their incorporation into clinical practice could significantly improve the effectiveness and safety of cancer therapies. Recent literature emphasizes the potential of these nanocomposites to overcome traditional chemotherapy limitations by providing targeted and efficient treatments with reduced side effects [56, 57]. However, further studies are needed to fully understand their mechanisms and optimize their use in clinical settings.

## Conclusion

Lung cancer remains is still a significant global health concern due to its high mortality rates and prevalence. Early detection and effective treatment are essential for improving patient outcomes. The integration of nanotechnology, particularly through the green synthesis of zinc/copper nanocomposites, offers promising therapeutic options for lung cancer treatment. Our research successfully synthesized Zn/Cu nanomaterials, which were characterized using UV-Vis, FT-IR, EDX, XRD, and FE-SEM techniques The nanoparticles took on a semi-spherical morphology with an average size of 49.37 nm. These nanocomposites showed significant antioxidant activity and inhibited the growth of human lung cancer cell lines SK-MES-1, A-549, and LK-2 with minimal toxicity to normal cells. The most promising results were seen in SK-MES-1 with an IC_50_ of 154.00 ± 1.83 µg/mL. While the present study reports promising results from in vitro cytotoxicity and antioxidant assays, an in vivo study is crucial to evaluate the bioavailability, toxicity, and overall therapeutic potential of the nanocomposite in a living organism to confirm safety and efficacy, our findings suggest that zinc/copper nanocomposites could be a powerful tool in combating lung cancer, potentially improving treatment outcomes by offering targeted and efficient therapeutic options. Ultimately, this research highlights the potential of nanotechnology to transform lung cancer treatment, providing new hope for enhanced survival rates and improved patient care.

## Data Availability

No datasets were generated or analysed during the current study.

## References

[1] Mejía-Méndez JL, López-Mena ER. E. Sánchez-Arreola, activities against lung cancer of biosynthesized silver nanoparticles: a review. Volume 11. Biomedicines; 2023. p. 389. 10.3390/biomedicines11020389 PMC9953519 36830926

[2] Wu S, Zhu W, Thompson P, Hannun YA. Evaluating intrinsic and non-intrinsic cancer risk factors. Nat Commun. 2018;9:3490. 30154431 10.1038/s41467-018-05467-z PMC6113228

[3] Gospodarowicz M, Brierley J, O’Sullivan B. Principles of cancer staging for clinical obstetrics and gynecology. Volume 29. Best Practice & Research Clinical Obstetrics & Gynaecology; 2015. pp. 767–75. 10.1016/j.bpobgyn.2015.05.003 26231930

[4] Zhang S, Li R, Jiang T, Gao Y, Zhong K, Cheng H, Chen X, Li S. Inhalable nanomedicine for lung cancer treatment. Smart Mater Med. 2024;5:261–80.

[5] Khalili M, Ebrahimi M, Fazlzadeh A, Moradkhani A, Jamali S. Evaluation of the survival rate and clinical outcome of nanodrug administration for the treatment of lung cancer: a systematic review and meta-analysis. Int J Sci Res Dent Med Sci. 2022;4:140–7.

[6] Semenova EA, Nagel R, Berns A. Origins, genetic landscape, and emerging therapies of small cell lung cancer. Genes Dev. 2015;29:1447–62. 26220992 10.1101/gad.263145.115 PMC4526731

[7] Herbst RS, Morgensztern D, Boshoff C. The biology and management of non-small cell lung cancer. Nature. 2018;553:446–54. 29364287 10.1038/nature25183

[8] Mukherjee A, Paul M, Mukherjee S. Recent progress in the theranostics application of nanomedicine in lung cancer. Cancers. 2019;11:597. 31035440 10.3390/cancers11050597 PMC6562381

[9] Yang D, Liu Y, Bai C, Wang X, Powell C.A. Epidemiology of lung cancer and lung cancer screening programs in China and the united States. Cancer Lett. 2020;468:82–7. 31600530 10.1016/j.canlet.2019.10.009

[10] Polański J, Świątoniowska-Lonc N, Kołaczyńska S, Chabowski M. Diet as a factor supporting lung cancer Treatment—A. Syst Rev Nutrients. 2023;15:1477. 10.3390/nu15061477 PMC10053575 36986207

[11] Norouzi M, Hardy P. Clinical applications of nanomedicines in lung cancer treatment. Acta Biomater. 2021;121:134–42. 33301981 10.1016/j.actbio.2020.12.009

[12] Nooreldeen R, Bach H. Current and future development in lung cancer diagnosis. Int J Mol Sci. 2021;22:8661. 34445366 10.3390/ijms22168661 PMC8395394

[13] Dzulkharnien NSF, Rohani R. A review on current designation of metallic nanocomposite hydrogel in biomedical applications. Nanomaterials. 2022;12:1629. 35630851 10.3390/nano12101629 PMC9146518

[14] Wu D, Zhou J, Creyer MN, Yim W, Chen Z, Messersmith PB, Jokerst JV. Phenolic-enabled nanotechnology: versatile particle engineering for biomedicine. Chem Soc Rev. 2021;50:4432–83. 33595004 10.1039/d0cs00908c PMC8106539

[15] Ahmad U, Ahmad Z, Khan AA, Akhtar J, Singh SP, Ahmad FJ. Strategies in development and delivery of nanotechnology based cosmetic products. Drug Res. 2018;68:545–52. 10.1055/a-0582-9372 29579762

[16] Enescu D, Cerqueira MA, Fucinos P, Pastrana LM. Recent advances and challenges on applications of nanotechnology in food packaging. A literature review. Food Chem Toxicol. 2019;134:110814. 31520669 10.1016/j.fct.2019.110814

[17] Li B, Moriarty TF, Webster T, Xing M. Racing for the surface: antimicrobial and interface tissue engineering. Springer; 2020.

[18] Sharma G, Kumar A, Naushad M, Pathania D, Sillanpää M. Polyacrylamide@ Zr (IV) vanadophosphate nanocomposite: ion exchange properties, antibacterial activity, and photocatalytic behavior. J Ind Eng Chem. 2016;33:201–8.

[19] Sharma G, Kumar A, Sharma S, Naushad M, Dwivedi RP, ALOthman ZA, Mola GT. Novel development of nanoparticles to bimetallic nanoparticles and their composites: A review. J King Saud University-Science. 2019;31:257–69.

[20] Padilla-Cruz A, Garza-Cervantes J, Vasto-Anzaldo XG, García-Rivas G, León-Buitimea A. Morones-Ramírez, synthesis and design of Ag–Fe bimetallic nanoparticles as antimicrobial synergistic combination therapies against clinically relevant pathogens. Sci Rep. 2021;11:5351. 33674678 10.1038/s41598-021-84768-8 PMC7935916

[21] Idris DS, Roy A. Synthesis of bimetallic nanoparticles and applications—an updated review. Crystals. 2023;13:637.

[22] Gomes Silva B, Pereira da Silva WF, Santos Soares JC, Lima Cavalcanti ID, De Souza IA, Cavalcanti L. Nanoparticles in the use of natural products for the treatment of lung cancer, DOI. 2019.

[23] Al-Attar AM, Alsalmi FA. Effect of Olea Europaea leaves extract on streptozotocin induced diabetes in male albino rats. Saudi J Biol Sci. 2019;26:118–28. 30622415 10.1016/j.sjbs.2017.03.002 PMC6318816

[24] Al-Snafi A. The nutritional and therapeutic importance of Olea europaea-a review. TMR Integr Med. 2023;7:e23030.

[25] Fanelli V, Mascio I, Falek W, Miazzi MM, Montemurro C. Current status of biodiversity assessment and conservation of wild Olive (Olea Europaea L. Subsp. Europaea Var. sylvestris). Plants. 2022;11:480. 35214813 10.3390/plants11040480 PMC8877956

[26] Carrión Y, Ntinou M, Badal E. Olea Europaea L. in the North mediterranean basin during the pleniglacial and the Early–Middle holocene. Q Sci Rev. 2010;29:952–68.

[27] Green PS. A revision of Olea L.(Oleaceae). Kew Bull. 2002;57:91–140.

[28] Arenas-Castro S, Gonçalves JF, Moreno M, Villar R. Projected climate changes are expected to decrease the suitability and production of Olive varieties in Southern Spain. Sci Total Environ. 2020;709:136161. 31905547 10.1016/j.scitotenv.2019.136161

[29] Guo Z, Jia X, Zheng Z, Lu X, Zheng Y, Zheng B, Xiao J. Chemical composition and nutritional function of Olive (Olea Europaea L.): A review. Phytochem Rev. 2018;17:1091–110.

[30] Scheffler A, Rauwald H, Kampa B, Mann U, Mohr F, Dhein S. Olea Europaea leaf extract exerts L-type Ca2 + channel antagonistic effects. J Ethnopharmacol. 2008;120:233–40. 18790040 10.1016/j.jep.2008.08.018

[31] Somova L, Shode F, Mipando M. Cardiotonic and antidysrhythmic effects of oleanolic and ursolic acids, Methyl maslinate and Uvaol. Volume 11. Phytomedicine; 2004. pp. 121–9. 10.1078/0944-7113-00329 15070161

[32] Hamdi HK, Castellon R. Oleuropein, a non-toxic Olive iridoid, is an anti-tumor agent and cytoskeleton disruptor. Biochem Biophys Res Commun. 2005;334:769–78. 16024000 10.1016/j.bbrc.2005.06.161

[33] Bhardwaj A, Singh S, Srivastava SK, Arora S, Reed E, Singh AP. Olive polyphenol, Hydroxytyrosol, exerts anti-tumor effects in prostate cancer cells by targeting Akt, STAT3 and AR signaling. Cancer Res. 2012;72:605–605.

[34] Talhaoui N, Trabelsi N, Taamalli A, Verardo V, Gómez-Caravaca A.M., Fernández-Gutiérrez A, Arraez-Roman D. Olea Europaea as potential source of bioactive compounds for diseases prevention. Stud Nat Prod Chem. 2018;57:389–411.

[35] Nicolì F, Negro C, Vergine M, Aprile A, Nutricati E, Sabella E, Miceli A, Luvisi A, De Bellis L. Evaluation of phytochemical and antioxidant properties of 15 Italian Olea Europaea L. cultivar leaves. Molecules. 2019;24:1998. 31137706 10.3390/molecules24101998 PMC6572269

[36] Kaskoos RA. Pharmacognostic specifications of leaves of Olea Europaea collected from Iraq. DOI: Original Article AJPCT [2][2]; 2013. pp. 153–60.

[37] Ayoub L, Hassan F, Hamid S, Abdelhamid Z, Souad A. Phytochemical screening, antioxidant activity and inhibitory potential of ficus carica and Olea Europaea leaves. Bioinformation. 2019;15:226. 31354199 10.6026/97320630015226 PMC6637399

[38] Ryan D, Antolovich M, Prenzler P, Robards K, Lavee S. Biotransformations of phenolic compounds in Olea Europaea L. Sci Hort. 2002;92:147–76.

[39] Taamalli A, Román DA, Zarrouk M, Segura-Carretero A, Fernández-Gutiérrez A. Classification of ‘chemlali’accessions according to the geographical area using chemometric methods of phenolic profiles analysed by HPLC–ESI-TOF–MS. Food Chem. 2012;132:561–6. 26434332 10.1016/j.foodchem.2011.10.070

[40] Suseela V, Nirmaladevi R, Pallikondaperumal M, Priya RS, Shaik MR, Shaik AH, Khan M, Shaik B. Eco-friendly Preparation of silver nanoparticles and their antiproliferative and apoptosis-inducing ability against lung cancer. Life. 2022;12:2123. 36556488 10.3390/life12122123 PMC9782107

[41] Hitkari G, Chowdhary P, Kumar V, Singh S, Motghare A. Potential of Copper-Zinc oxide nanocomposite for photocatalytic degradation of congo red dye. Clean Chem Eng. 2022;1:100003.

[42] Mahdavi B, Paydarfard S, Rezaei-Seresht E, Baghayeri M, Nodehi M. Green synthesis of NiONPs using trigonella subenervis extract and its applications as a highly efficient electrochemical sensor, catalyst, and antibacterial agent. Appl Organomet Chem. 2021;35:e6264.

[43] Li J, Mahdavi B, Baghayeri M, Rivandi B, Lotfi M, Zangeneh MM, Zangeneh A, Tayebee R. A new formulation of Ni/Zn bi-metallic nanocomposite and evaluation of its applications for pollution removal, photocatalytic, electrochemical sensing, and anti-breast cancer. Environ Res. 2023;233:116462. 37352956 10.1016/j.envres.2023.116462

[44] Shu M, Mahdavi B, Balčiūnaitienė A, Goorani S, Mahdavi AA. Novel green synthesis of Tin nanoparticles by medicinal plant: chemical characterization and determination of cytotoxicity, cutaneous wound healing and antioxidant properties. Micro Nano Lett. 2023;18:e12157.

[45] Zhang Y, Mahdavi B, Mohammadhosseini M, Rezaei-Seresht E, Paydarfard S, Qorbani M, Karimian M, Abbasi N, Ghaneialvar H, Karimi E. Green synthesis of NiO nanoparticles using calendula officinalis extract: chemical charactrization, antioxidant, cytotoxicity, and anti-esophageal carcinoma properties. Arab J Chem. 2021;14:103105.

[46] Girigoswami A, Girigoswami K. Potential applications of nanoparticles in improving the outcome of lung cancer treatment. Genes. 2023;14:1370. 37510275 10.3390/genes14071370 PMC10379962

[47] Soni A, Bhandari MP, Tripathi GK, Bundela P, Khiriya PK, Khare PS, Kashyap MK, Dey A, Vellingiri B, Sundaramurthy S. Nano-biotechnology in tumour and cancerous disease: A perspective review. J Cell Mol Med. 2023;27:737–62. 36840363 10.1111/jcmm.17677 PMC10002932

[48] Loza K, Heggen M, Epple M. Synthesis, structure, properties, and applications of bimetallic nanoparticles of noble metals. Adv Funct Mater. 2020;30:1909260.

[49] Dykman LA, Khlebtsov NG. Uptake of engineered gold nanoparticles into mammalian cells. Chem Rev. 2014;114:1258–88. 24279480 10.1021/cr300441a

[50] Kopp M, Kollenda S, Epple M. Nanoparticle–protein interactions: therapeutic approaches and supramolecular chemistry. Acc Chem Res. 2017;50:1383–90. 28480714 10.1021/acs.accounts.7b00051

[51] Zhou B, Chen M, Hao Z, Li L, Zhang Y, Fang B, Shao M, Ren G, Wang K, Liu H. Zinc-copper bimetallic nanoplatforms trigger photothermal-amplified Cuproptosis and cGAS-STING activation for enhancing triple-negative breast cancer immunotherapy. J Nanobiotechnol. 2025;23:137. 10.1186/s12951-025-03186-4 PMC11849371 39994712

[52] Zadeh FA, Bokov DO, Salahdin OD, Abdelbasset WK, Jawad MA, Kadhim MM, Qasim MT, Kzar HH, Al-Gazally ME, Mustafa YF. Cytotoxicity evaluation of environmentally friendly synthesis copper/zinc bimetallic nanoparticles on MCF-7 cancer cells, rendiconti lincei. Scienze Fis E Naturali. 2022;33:441–7. 10.1007/s12210-022-01064-x PMC8936039 35342535

[53] Fereydouni P, Al Mohaddesin A, Khaleghi S. Targeted biocompatible Zn-metal–organic framework nanocomposites for intelligent chemotherapy of breast cancer cells. Sci Rep. 2024;14:18311. 39112669 10.1038/s41598-024-69457-6 PMC11306755

[54] Li M, Zhang Z, Yu Y, Yuan H, Nezamzadeh-Ejhieh A, Liu J, Pan Y, Lan Q. Recent advances in Zn-MOFs and their derivatives for cancer therapeutic applications. Mater Adv. 2023;4:5050–93.

[55] Abdolmaleki S, Aliabadi A, Khaksar S. Unveiling the promising anticancer effect of copper-based compounds: A comprehensive review. J Cancer Res Clin Oncol. 2024;150:213. 38662225 10.1007/s00432-024-05641-5 PMC11045632

[56] Yassin MT, Al-Otibi FO, Al-Sahli SA, El-Wetidy MS, Mohamed S. Metal oxide nanoparticles as efficient nanocarriers for targeted cancer therapy. Volume 16. Cancers: Addressing Chemotherapy-Induced Disabilities; 2024. p. 4234. 10.3390/cancers16244234 PMC11674168 39766133

[57] Andoh V, Ocansey DKW, Naveed H, Wang N, Chen L, Chen K, Mao F. The advancing role of nanocomposites in cancer diagnosis and treatment. Int J Nanomed. 2024;19:6099–126. 10.2147/IJN.S471360 PMC11194004 38911500

